# Obituary

**Published:** 1848-10

**Authors:** 


### Obituary.

J. P. HUNT, D. D. S.—As a painful duty, we announce the death of this gentleman, for several years past a resident of Indianopolis, Ind. At the late meeting of the Mississippi Valley Association of Dental Surgeons, we confidently anticipated the cordial greetings of our much esteemed professional brother, and when our worthy President at our first session made known his intention to resign, on account of having withdrawn from the profession, our mind naturally turned to Dr. Hunt as one of our Vice Presidents to occupy the chair. When the above announcement was made known, we need scarcely add, that it was received with deep regret by every member of the Society, as we know it must have been by his numerous friends in the city of his residence.

We have known the Doctor for several years, as an honest, upright, and indefatigable dental practitioner, fully bent upon acquiring and putting to use, such knowledge as might elevate the standard of dental attainment, and be of use professionally to his patients.

We are assured his reputation and practice increased in a full ratio with the rapid improvement and growth of the metropolis of our neighbor state.

Some time will elapse before another so fully secures the confidence of the people of that city and adjoining towns.—CIN. ED.

